# Expression profiling by high-throughput sequencing reveals GADD45, SMAD7, EGR-1 and HOXA3 activation in Myostatin (MSTN) and GDF11 treated myoblasts

**DOI:** 10.1590/1678-4685-GMB-2023-0304

**Published:** 2024-07-15

**Authors:** Platon Braun, Malik Alawi, Ceren Saygi, Klaus Pantel, Amy J. Wagers

**Affiliations:** 1 Harvard University, Department of Stem Cell and Regenerative Biology, Cambridge, MA, United States.; 2 University Medical Center Hamburg-Eppendorf, Department of Tumor Biology, Hamburg, Germany.; 3 University Medical Center Hamburg-Eppendorf, Department of Oncology, Hematology and Bone Marrow Transplantation with section Pneumology, Hamburg, Germany.; 4 University Medical Center Hamburg-Eppendorf, Bioinformatics Core, Hamburg, Germany.; 5 Joslin Diabetes Center, Inc., Boston, MA, United States.

**Keywords:** GDF11, Myostatin, RNA sequencing, myoblasts, gene expression

## Abstract

Growth differentiation factor 11 (GDF11) and myostatin (MSTN/GDF8) are closely related members of the transforming growth factor β (TGFβ) superfamily, sharing structural homology. Despite these structural similarities, recent research has shed light on the distinct roles these ligands play within muscle tissue. This study aims to uncover both the differences and similarities in gene expression at the transcriptome level by utilizing RNA sequencing. We conducted experiments involving five distinct groups, each with three biological replicates, using C2C12 cell cultures. The cells were subjected to high-throughput profiling to investigate disparities in gene expression patterns following preconditioning with either GDF11 or MSTN at concentrations of 1 nM and 10 nM, respectively. In addition, control groups were established. Our research revealed concentration-dependent gene expression patterns, with 38 genes showing significant differences when compared to the control groups. Notably, GADD45, SMAD7, EGR-1, and HOXA3 exhibited significant differential expression. We also conducted an over-representation analysis, highlighting the activation of MAPK and JNK signaling pathways, along with GO-terms related to genes that negatively regulate metabolic processes, biosynthesis, and protein phosphorylation. This study unveiled the activation of several genes not previously discussed in existing literature whose full biological implications are yet to be determined in future research.

## Introduction

Considering demographic changes in today’s society, preserving muscle function and its regenerative potential are key goals in morbidity and mortality prevention in the aging population (Li et al., 2018; Papadopoulou 2020). In conjunction with the high costs, age-related muscle dysfunction leads to a significant reduction of life quality, progressive weakness, and disability (Janssen et al., 2004; Siparsky et al., 2014; Trombetti et al., 2016). A wide variety of diseases manifesting in muscle dysfunction, with no satisfactory treatment alternatives and no curative therapy options, further increase the clinical urgency and growing interest in muscle aging and regeneration research. 

Cytokines of the transforming growth factor β (TGFβ) family contain more than 30 biochemically related but functionally distinct ligands, which are known to play a crucial role in the regulation of developmental patterning, cellular proliferation and differentiation, and the maintenance of tissue homeostasis (Walker et al., 2016; Morikawa et al., 2016). Among others, Growth Differentiation Factor 11 (GDF11) and MSTN are structurally closely related TGFβ family members and share 90% homology in their mature active regions (Walker *et al.*, 2016). 

While MSTN is expressed primarily in skeletal muscles, GDF11 is expressed broadly in numerous tissues (McPherron et al., 1997; Nakashima et al., 1999; Gamer et al., 1999) and has been subject to conflicting reports regarding its role in muscle regeneration, aging-process and cancer. Some studies also managed to demonstrate tumor suppressive properties of GDF11 in the Breast and Liver cancer (Bajikar et al., 2017; Gerardo-Ramírez et al., 2019). Additionally, its expression seems to correlate negatively with tumor stage in colorectal cancer and positively with the survival rate in patients with pancreatic cancers (Yokoe et al., 2007; Liu et al., 2018). Prior work also implicates the role GDF11 plays in aging-related phenotypes in the heart, skeletal muscle, and brain (Loffredo et al., 2013; Katsimpardi et al., 2014; Sinha et al., 2014; Egerman et al., 2015; Poggioli et al., 2016; Walker et al., 2016; Kraler et al., 2023) and its expression in epithelial cells of the developing stomach and duodenal epithelium (Harmon et al., 2004).

Despite the high structural similarity between GDF11 and MSTN, mutations in these molecules lead to very different phenotypical manifestations, indicating divergent functionality across numerous tissues. Unlike genetic deficiency of MSTN, which leads to hypermuscularity (McPherron et al., 1997; McPherron and Lee, 1997), homozygous deletion of GDF11 generates defects in axial skeletal patterning and organ development, subsequently leading to perinatal lethality in GDF11-null mice (McPherron *et al.*, 1999).

GDF11 and MSTN are synthesized as precursors and undergo proteolytic processing to produce biologically active mature ligands. Both proteins bind to activin type II receptors (ACVR2A or ACVR2B), and recruit activin receptor-like kinase 4 and/or 5 (ALK4/ ALK5) forming a heteromeric receptor complex to induce downstream intracellular SMAD2/3 mediator pathway signaling via phosphorylation. GDF11 also showed the ability to activate SMAD1/5/9 phosphorylation through the utilization of the ALK1 receptor in different tissues, leading to proliferation and differentiation. Additionally, even though both, GDF11 and MSTN, are able to activate similar receptor types as well as ALK7 receptors, GDF11 has shown to initiate a stronger signal, implicating a higher binding affinity (Walker et al., 2016; Walker *et al.*, 2017). 

Despite all similarities in protein sequence or receptor utilization, along with signaling pathways, several studies demonstrated that these two ligands may have different functions across numerous tissues and among others in muscle cells (McPherron et al., 1997; Nakashima et al., 1999; Walker et al., 2016). It has been shown that GDF11 and MSTN are able to initiate some unique activity on muscle, aside from its activation of known (ActRII/ALK/SMAD) signaling. Previous studies suggest that there are noncanonical pathways for activating other non-SMAD proteins, such as p38 MAPK, ERK, and JNK, as well as different mechanisms of modulation of the activity of GDF11 and MSTN through extracellular binding proteins. These extracellular binding proteins, which typically function as antagonists, include follistatin (FST), follistatin-like 3 (FSTL3/FLRG), growth and differentiation factor-associated serum protein 1 (GASP1), GASP2, latent TGF-β binding protein 3 (LTBP3), and decorin (DCN) (Philip et al., 2005; Yang et al., 2006; Wang et al., 2014; Egerman et al., 2015; Biesemann et al., 2015; Walker *et al.*, 2016). 

Believed to have very similar functions, the question, of why those two structurally highly homologous proteins have different secretion patterns and effects within various cell types and additionally vary in concentration throughout the lifespan, remains a high priority and subject to ongoing investigations. In this study, we aimed to investigate and expose differences in gene activation patterns, for the first time utilizing RNA sequencing in GDF11 and MSTN-treated C2C12 cell cultures, a subclone from a myoblast cell line, originally isolated by Yaffe and Saxel at the Weizmann Institute of Science in Israel (Yaffe and Saxel, 1977).

Due to the fact that MSTN-null mice survived to adulthood, whereas GDF11-null mice died shortly after birth, a great number of studies used recombinant GDF11 and/ or MSTN proteins in order to reveal the exact function of these ligands. The role of MSTN in satellite cells has been the subject of controversial results, ranging from an inhibitor of C2C12 myoblast proliferation in early studies (Thomas et al., 2000; Taylor et al., 2001), to Rodgers et al. (2014) arguing that recombinant MSTN stimulates C2C12 proliferation. Additionally, the source of the recombinant protein could be an outcome-determining factor on itself (Rodgers *et al.*, 2014).

Sinha et al. (2014) demonstrated that the aged mice treated with recombinant GDF11 protein injections displayed improved metrics in skeletal muscle strength, endurance, muscle regeneration, and even myofibrillar and mitochondrial morphology (Sinha *et al.*, 2014). Further study in fish showed the application of GDF11 recombinant proteins being able to boost antioxidant enzyme activity in muscle, prolonging the lifespan (Zhou et al., 2019). While several studies failed to reproduce those results or even showed conflicting outcomes like impaired muscle regeneration and satellite cell expansion (Egerman et al., 2015; Hinken et al., 2016) our study aimed to provide insights and deeper understanding of the particular function of GDF11 and MSTN in muscle tissue and further outline possible target genes for disease monitoring and interventions. 

## Material and Methods

### RNA sequencing preparation

C2C12 cells, a subclone of the mouse myoblast cell line established by D. Yaffe and O. Saxel (ATCC CRL-1772), biosafety level 1(biosafety classification is based on U.S. Public Health Service Guidelines), were acquired from Thermo Fisher Scientific. Initial experiments were performed at the Harvard University Department of Stem Cell and Regenerative Biology at Wagers Laboratory, Cambridge, MA, US. C2C12 cells are immortalized mouse myoblast cells with very short replication time that can be rapidly differentiated into functional skeletal or cardiac muscle cells (McMahon et al., 1994).

### C2C12 cell culturing protocol

C2C12 cells from frozen stock were cultured in a 50 ml flask, and 10 ml special media containing 445 ml of 1 x DMEM - Dulbecco’s Modified Eagle Medium, 50 ml FBS - Fetal Bovine Serum, and 5ml Pen-Strep - Penicillin-Streptomycin (10,000 units penicillin and 10 mg streptomycin/mL). Cultures were split when they reached 80% confluency, approximately every 48 h. 

Activation of 2/3 SMAD and/or p-SMAD (sc-6032, Lot#G2214 and sc-11769, Lot# H2014, Antibodies, Santa Cruz Biotechnology) pathways was confirmed using the Protein Simple Wes system. 

### mRNA extraction

All experimental groups had three biological replicates (A-C), with a total number of 15 samples. C2C12 cells were incubated in the growth media until approximately 70% confluency was reached, then washed with PBS and incubated in a starvation media for 3 hr. The relatively short starvation allowed to avoid reduced cell survival and increased apoptosis as a reaction to prolonged starvation. It also provided enough time for the adaptation processes in cell metabolism and the induction of cell cycle synchronization. 

Starvations media contained 5 mL Pen-Strep = Penicillin-Streptomycin (10,000 units penicillin and 10 mg streptomycin/mL), 495 mL DMEM, and 200 uL FBS (0.02%).

Recombinant pure proteins GDF11, and MSTN (PEPROTECH, USA, REF #120-11 and #120-00) were prepared using following buffer: 10 mM HCL, 0.1% protease free BSA, and PBS. Subsequently, starvation media was changed, and the cultures were divided into five experimental groups and co-incubated with recombinant proteins GDF11, and MSTN at concentrations of 1 nM and 10 nM, as well as “buffer only” vehicle group, for the duration of one hour. Based on previous studies the concentration of both proteins varied across different tissues and during the lifespan. Additionally, there is a known difference in receptor affinity for both proteins. To account for both factors in our experiments, we utilized two different concentrations for each protein.

Plates were twice washed in ice cold PBS, then total RNA was extracted from all 20 experimental samples using TRIzol Reagent Protocol (Thermo Fisher: Pub. No. MAN0001271). The concentration of RNA was confirmed using NanoDrop and Qubit. BioAnalzyer was used to assess the quality of the probes. RNA Integrity Number (RIN) values ranged from 9.9 - 10. 

### Library preparation

Sequencing was performed at Bauer Core Facility, Harvard University, Cambridge, USA. Libraries were prepared using a SciClone G3 NGSx workstation (Perkin Elmer) using the Kapa mRNA HyperPrep kit (Roche Sequencing). Polyadenylated mRNAs were captured using oligo-dT-conjugated magnetic beads (Kapa mRNA HyperPrep kit, Roche Sequencing) from 500 ng of total RNA on a Perkin Elmer SciClone G3 NGSx automated workstation. Poly-adenylated mRNA samples were immediately fragmented to 200-300 bp using heat and magnesium. First-strand synthesis was completed using random priming, followed by second-strand synthesis and A-tailing. A dUTP was incorporated into the second strand to allow strand-specific sequencing of the library. Libraries were enriched and indexed using nine cycles of amplification (Kapa mRNA HyperPrep kit, Roche Sequencing) with PCR primers, which included dual 8 bp index sequences to allow for multiplexing (IDT for Illumina unique dual 8 bp indexes). Excess PCR reagents were removed through magnetic bead-based cleanup using KAPA Pure magnetic beads on a Sciclone G3 NGSx workstation (Perkin Elmer). The resulting libraries were assessed using a 4200 TapeStation (Agilent Technologies) and quantified by qPCR (Roche Sequencing). Libraries were pooled and sequenced using paired-end, 75 bp reads. On average, 27.4 M reads were obtained per library (SD: 3.3 M).

### RNA sequencing data analysis

Sequence reads were processed with fastp (v0.20.1) to remove sequences of sequencing adapters and low quality (Phred quality score below 15) sequences from the 3’-end of the sequence reads (Chen et al., 2018). Hereafter, reads were aligned to the mouse reference assembly (GRCm39.104) using STAR (v2.7.9a) (Dobin et al., 2013). Differential expression was assessed with DESeq2 (Love et al., 2014). A gene was considered to be significantly differentially expressed if the corresponding false discovery rate (FDR) did not exceed a value of 0.05 and the absolute value of the logarithmic fold change (log2FC) was 1 or higher. The detection of GO-terms over-represented in sets of differentially expressed genes was performed using GOrilla (database version: Mar 6, 2021) (Eden et al., 2009) in combination with the Gene Ontology database (Ashburner et al., 2000).

## Results

High-throughput gene expression profiling was conducted on C2C12 cell cultures, comprising five distinct experimental groups, each with three biological replicates. Within each group, cells were pre-treated with either GDF11 or MSTN at concentrations of 1 nM and 10 nM. Control groups treated with a vehicle were also included in the study.

**Figure 1 -:**
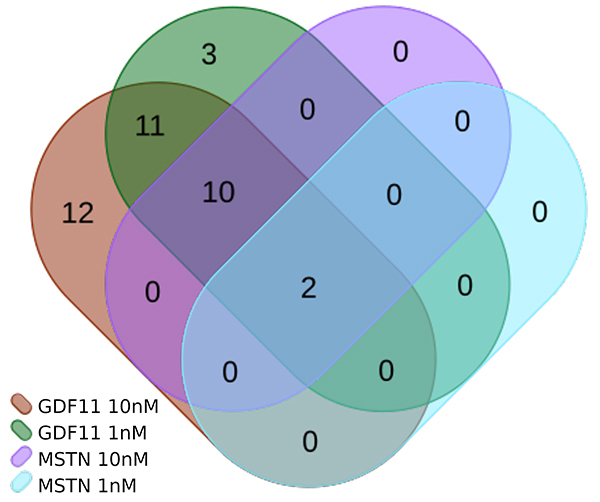
Intersections of genes that are significantly differentially expressed in comparison to the control. The two respectively 12 genes that are differentially expressed between MSTN and the control are also differentially expressed when GDF11 is compared to the control. Contrary to the general trend, there appear to be three genes that are differentially expressed solely between GDF11 1 nM and the control. However, DUSP6 and TNFSF15 both satisfy the FDR criterion and miss the foldchange criterion (|log2FC| >= 1) in GDF11 10 nM compared to control only narrowly with log2 foldchanges of -0.9 and 0.97.

We conducted separate assessments of genes that exhibit upregulation or downregulation when compared to the control group. Although we did not anticipate or investigate linear correlations, we observe clear distinctions among the treatment groups (Figure S1). Thirty-five genes were significantly differentially expressed in the comparison of GDF11 10 nM and the control. 28 of these were higher expressed in GDF11 10 nM, whereas seven were higher expressed in the control. In the comparison of GDF11 1 nM, MSTN 10 nM and MSTN 1 nM 26, 12, and 2 genes were differentially expressed respectively, whereas the genes differentially expressed in the comparisons including MSTN were wholly contained in the sets of genes of the comparisons including GDF11 (Figure 1). In addition we performed direct comparisons between all treatment groups. In the comparison of GDF11 1 nM and MSTN 1 nM, three genes (Smad7, Wnt9a and Hbegf) are significantly differentially expressed. In the comparison GDF11 10 nM and MSTN 1 nM, there are six differentially expressed genes (Smad7, Wnt9a, Hbegf, Bhlhe40, Dusp2 and CCn2). No differentially expressed genes could be detected in the other direct comparisons.

**Figure 2 -:**
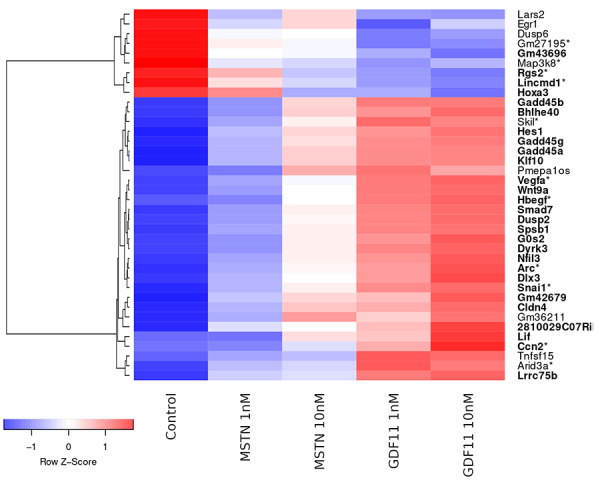
Row Z-Scores of the normalized expression of all 38 genes that were significantly differentially expressed in at least one comparison. The expression of most genes increases (n=24) or decreases (n=4) continuously from the Control over MSTN 1 nM, MSTN 10 nM and GDF11 1 nM to GDF11 10 nM. The corresponding gene symbols are printed in bold. Additionally, genes which were exclusively differentially expressed in both of the two GDF10 groups are marked with an asterisk.

While at first, it might seem that there are 12 or 13 genes that play a role solely in GDF11 10 nM and GDF11 1 nM, respectively, a closer look reveals that gene expression instead undergoes a gradual change from control to MSTN 1 nM, MSTN 10 nM, GDF11 1 nM, and then GDF11 10 nM (Figure 2). The comparatively small changes between GDF11 1 nM and GDF11 10 nM could indicate that, in the case of GDF11, a concentration of 1 nM already produces an effect that can be increased at most slightly at higher concentrations. Apart from the concentration dependence described above, no expression differences suggesting different regulatory mechanisms of GDF11 and MSTN were observed.

Over-representation analysis (ORA) was performed based on the comparison of GDF11 10 nM and the control. Genes corresponding with the Gene Ontology (GO) -terms associated with MAPK activation (GO:1900745, GO:0000185, GO:0032874, GO:0046330, GO:0043410) and JNK (GO:0046330) were significantly enriched and displayed the smallest FDR. Furthermore, the analysis showed GO-terms correlated to negative regulation of metabolic processes (e.g., GO:0010605, GO:0009892), biosynthesis activity (e.g., GO:0009890), and protein phosphorylation (e.g., GO:0001933).

The sequence reads have been submitted to the European Nucleotide Archive (ENA) as part of the study PRJEB57932. For accession numbers please refer to Table S1.

## Discussion

To our knowledge, this is the first study to report gene expression pattern comparison between GDF11 and MSTN in C2C12 cell culture using RNA sequencing (RNA-seq) technology. In previous microarray analysis of hSkMDCs treated with GDF11 or MSTN performed by Egerman et al. (2015) the human primary muscle cells were exposed to a 24-hour stimulation with 300 ng/ml of MSTN, GDF11, or buffer alone (serving as a negative control). Subsequent analysis of the log fold change in gene expression demonstrated that GDF11 and MSTN induced very similar, insignificantly different, genes expression patterns (Egerman *et al.*, 2015). However, RNA-seq provides counts of aligned sequence reads, resulting in a very broad dynamic range, improving the possibility of detecting rare transcripts and conclusively increasing its sensitivity and accuracy. Additionally, RNA-seq does not use probes or primers, therefore, the data suffer from much lower biases allowing more robust results. Mentioned above also results in RNA-seq outrunning microarrays for their high degree of reproducibility (Wang et al., 2009; Wilhelm and Landry, 2009; Zhao et al., 2014; Liu et al., 2015; Rao et al., 2019). Rather than restricting the comparison of the gene activation pattern of GDF11 and MSTN directly, we took the approach of specifically looking into the expression patterns in comparison to the vehicle controls, which allowed us to further eliminate noise, increasing the sensitivity of our experiment. 

These are two structurally very similar and closely related ligands that naturally circulate in the blood in their inactive form. Recent studies demonstrate that the molar concentration of circulating MSTN seemed to be around 500 times higher than that of GDF11 (Rodgers and Eldridge, 2015). Furthermore, being ~90% identical in their mature domains, there is only ~52% identity in their pro-domains (Walker et al., 2017), leading to the conclusion that their activity depends on structural changes, previously described regulatory mechanisms and receptor utilization in target tissue (Zimmers et al., 2002; Walker *et al.*, 2016). Walker *et al.* (2017) described MSTN as having lower binding affinity to all type 1 receptors, conclusively GDF11 inducing a greater SMAD3-dependent signal (Walker *et al.*, 2017). Although the biological consequences of the described differences remain to be determined, our study clearly underlined differences in binding affinity, indirectly confirming these findings. As visualized in Fig. 2, the gradual increase in signal correlates with the respective increase in ligand concentration, with MSTN requiring higher concentration to induce comparable strength in downstream activation.

Upon utilization of the string network analysis, we could demonstrate that there are described relationships between 38 differentially expressed genes in GDF11/ MSTN vs. controls, with a subset of genes clustered in groups. Even though our study did not specifically aim to explain the function of the 38 genes found to be significantly differentially expressed, there is a rationale for deriving possible functions based on clustered groups and already published data. Here we present a subset of these genes, that might offer a deeper understanding of functioning of these two ligands. In our belief, supported by previous studies, these selected genes represent the most promising targets for in-depth analysis, which could reveal unknown functions and interactions of these two ligands in future investigations.

### Gadd45 α/ β/ γ network

GADD45 (growth arrest and DNA-damage inducible) is a gene family of three highly homologous small nuclear proteins: GADD45α, GADD45β, and GADD45γ, that have been linked to regulation of many cellular functions including DNA repair, cell cycle control, genotoxic stress, as well as senescence and apoptosis. Due to the pro-apoptotic activities, GADD45 is believed to play an essential role in oncogenesis, functioning as a tumor suppressor. All three members of the family are found in muscle tissue with GADD45β (also known as Myd118) being the least abundant of the three (Tamura et al., 2012). 

MSTN is a potent negative regulator of skeletal muscle growth and its inactivation, as previously described, induces skeletal muscle hypertrophy, while its overexpression leads to muscle atrophy. The role of GDF11 in muscle has been subject to conflicting reports, and its function in muscle tissue is still to be determined. Interestingly our results show equal dose-dependent activation of the GADD45 network with both ligands, pointing towards a similar function. Bullard et al. (2016) and Ebert et al. (2012) both described the role of GADD45α in muscle atrophy either by activating stress-inducible, pro-atrophy transcription factor ATF4 or the interaction with MAP3K4, a mitogen-activated protein kinase complex (Ebert *et al.*, 2012; Bullard *et al.*, 2016). Over-representation of GO-terms linked to negative regulation of metabolic processes (e.g., GO:0010605, GO:0009892), biosynthesis activity (e.g., GO:0009890), as well as protein phosphorylation (e.g., GO:0001933), further supports the theory of negative regulation of skeletal muscles through GADD45 network (Figure S2). To our knowledge, there is no previous reporting on GADD45 activation through GDF11/ MSTN, which could be a missing link between these two ligands and their activity in muscles and an exciting study subject for future investigations. 

### SMAD7

Forbes et al. (2006) described Smad7 as being involved in the auto-regulation of the MSTN promotor by the negative feedback loop (Forbes *et al.*, 2006). The auto-regulation triggered by MSTN appears to be dose-dependent, as shown by our results, demonstrating a correlation between expression averages and the MSTN concentration used (Forbes *et al.*, 2006). Furthermore, according to a more recent study, the loss of Smad7 leads to various changes in muscle physiology, resulting in decreased muscle mass, impaired force generation, delayed recovery from injury, and many more (Cohen et al., 2015). Conclusively, a distracting negative loop leads to adverse effects of MSTN overexpression in muscle tissue. 

Moreover, according to our results, GDF11 triggered an even stronger SMAD7 response leading to the conclusion that this same protein may be involved in regulating GDF11 expression. Previous studies explored different regulatory mechanisms of GDF11/ MSTN activity, including Growth and differentiation factor (GDF) -associated serum protein-1 (GASP-1) and GASP-2, an inhibitor of mature MSTN and GDF11 (Lee and Lee, 2013; Walker et al., 2016). However, SMAD7 has not been previously reported in conjunction with GDF11 and muscle tissue, warranting further investigation.

### EGR1

Early growth response transcription factor (EGR1) has been subject to reports linked to its negative role in cardiovascular disease progression (Khachigian, 2021), profibrotic response, and physiologic and pathological connective tissue remodeling (Bhattacharyya et al., 2011). Given its manifold role in various tissue, our study discovered the activation of this transcript being weaker with GDF11 compared to MSTN. 

MSTN has been shown to directly regulate fibrosis in muscle tissue by stimulating the proliferation of muscle fibroblasts through activation of Smad, p38 MAPK, and Akt pathways (Li et al., 2008). Inhibition of MSTN was demonstrated to reverse muscle fibrosis by reducing fibroblasts’ resistance to the apoptosis (Li *et al.,* 2012). The utilization of GO-term analysis using the ORA, demonstrated positive activation of the p38MAPK and MAP3K cascade, indirectly confirming the results of Li et al. (2008) and pointing towards fibrosis regulation through EGR-1 triggered p38MAPK signaling (Figure S2). Having both physiologic and pathologic functions, EGR-1 should be further studied towards its role in MSTN-provided muscle tissue fibrosis and its activation through GDF11. 

### HOXA3

Gradual inhibition of the Hoxa3 genes was noted in both GDF11 and MSTN-stimulated C1C12 cells in our study. It also seemed to be inversely correlated with concentrations in both ligands. Turner et al. (2020) did great work describing epigenetic changes in skeletal muscle tissue by investigating the methylation status of various subsets of genes in their recent study. Interestingly, Hoxa3 showed an inverse relationship between gene expression and methylation, demonstrating significantly downregulated expression through hypermethylation in aged cells. Further investigations by this group have also determined that increased levels of endurance exercises were able to reverse methylation and increase gene expression of Hoxa3, practically reversing age-related epigenetic changes in muscle cells (Turner *et al.*, 2020). Another study concluded that GDF11 could help to prevent cardiomyocyte pyroptosis (inflammation-dependent type of programmed cell death) via GDF11/Smad2/3/HOXA3/NLRP3 (Nucleotide-binding oligomerization domain-like receptor pyrin domain-containing 3) signaling pathway, by leading to overexpression of Hoxa3 with subsequent inhibition of NLRP3 (Li et al., 2020). Our study could not identify an increase in HOXA3 gene expression upon exposure to GDF11/ MSTN. In summary, there is little known about GDF11/ MSTN triggered HOXA3 activity in skeletal muscle tissue, therefore, it is our belief that our results should prompt further investigation that might uncover the additional mechanism of action of these two ligands.

One significant limitation in our experimental design may stem from the duration of the experiment. Specifically, the treatment with GDF proteins was conducted for the period of one hour, with sequential harvesting for further analysis. However, even though some biological processes, particularly those related to differentiation, could need extended time frames, the main goal our experiment was the genes expression patterns analysis. Satisfying time for these processes was assured by confirmation of SMAD activation utilizing Western Blot.

Another limitation of our study that is to be mentioned is the absence of validation with qPCR. Past experiences with microarrays suggested the necessity of such validation of genome-scale expressions using RT-qPCR analysis in order to mitigate issues with reproducibility and bias (Zhang et al., 2006; Balázsi and Oltvai, 2007). Confirmation of RNA sequencing results with quantitative Reverse Transcription Polymerase Chain Reaction (qRT-PCR) is a common practice in molecular biology and genomics research, and it serves a valuable purpose in many cases. However, there are situations where confirmation with qRT-PCR may not be necessary or may not provide substantial additional benefits. High-throughput RNA sequencing platforms have become increasingly accurate and reliable. These platforms are designed to generate precise gene expression data, and many researchers have confidence in the quality of their results, especially when using well-established protocols and bioinformatics pipelines. A number of studies have specifically addressed the correlation between results obtained with RNA seq and qPCR, showing similar results as those published by Everaert et al. (2017). Furthermore, as Coenye T. outlined in his recent article the RNA-seq methods and data analysis approaches are robust and therefore do not always require confirmation through other approaches or methods such as RT qPCR (Coenye, 2021). 

Even though our study demonstrated similar direct signaling of GDF11 and MSTN, and confirmed the results of the microarray analysis performed by Egerman et al. (2015), it is still possible that GDF11 and MSTN might have some unique divergent activity on myoblasts and muscle tissue. As several studies have already stressed, the activity of both molecules in different tissues is not only determined through the activity of the mature ligand itself but also through the cleaving activity of proteases that cleave off the pro domains and additionally by modulation and inhibition by different extracellular binding proteins, as well as the availability of specific binding domains (Kondás et al., 2008; Walker et al., 2016). Described mechanisms could, technically speaking, change the outcome of the experiment in vivo since using active ligands and cell culture does not allow any further modification steps and changes that might happen in tissue and be crucial for these ligands’ function. 

Furthermore, the actual source of the ligands used was previously already described as a possible important factor that could alter experimental results (Rodgers et al., 2014), implicating that there is an actual rationale in determining expression patterns utilizing ligands from various sites and comparing the downstream activation. Assuming that the source of the ligands could play a vital role gives a high probability of conflicting results if performing experiments with ligands from different vendors. 

## Conclusions

In conclusion, in part contracting studies highlight the need for further investigation to fully delineated and uncover the functions of GDF11 and MSTN in muscle tissue. Our study did not reveal any statistical difference in gene expression patterns between these two ligands. ORA confirmed the activation of the non-canonical pathway by the enrichment of GO-terms for MAPK (GO:0043410) and JNK activation (GO:0046330). Furthermore, we were able to show similar activation patterns and outline novel relationships between genes not yet described in connection with GDF11/ MSTN in muscle tissue, pointing out important questions for future investigations.

## Supplementary material

The following online material is available for this article:

Figure S1 - Boxplots.

Figure S2 - Vulcano plots.

Table S1 - Accession Numbers.
