# Supplementary material for: Expression profiling by high-throughput sequencing reveals GADD45, SMAD7, EGR-1 and HOXA3 activation in Myostatin (MSTN) and GDF11 treated myoblasts
Source: Genet Mol Biol. 2024 Jul 15;47(2):e20230304. doi: 10.1590/1678-4685-GMB-2023-0304 (PMC11256782; doi:10.1590/1678-4685-GMB-2023-0304)
Supplement: Figure S1 - [file 1415-4757-GMB-47-02-e20230304-s1.pdf]

Genetics and Molecular Biology
https://doi.org/10.1590/1678-4685-GMB-2023-0304

Supplementary Material to "Expression profiling by high-throughput sequencing reveals GADD45, SMAD7, EGR-1 and HOXA3 activation in Myostatin (MSTN) and GDF11 treated myoblasts"

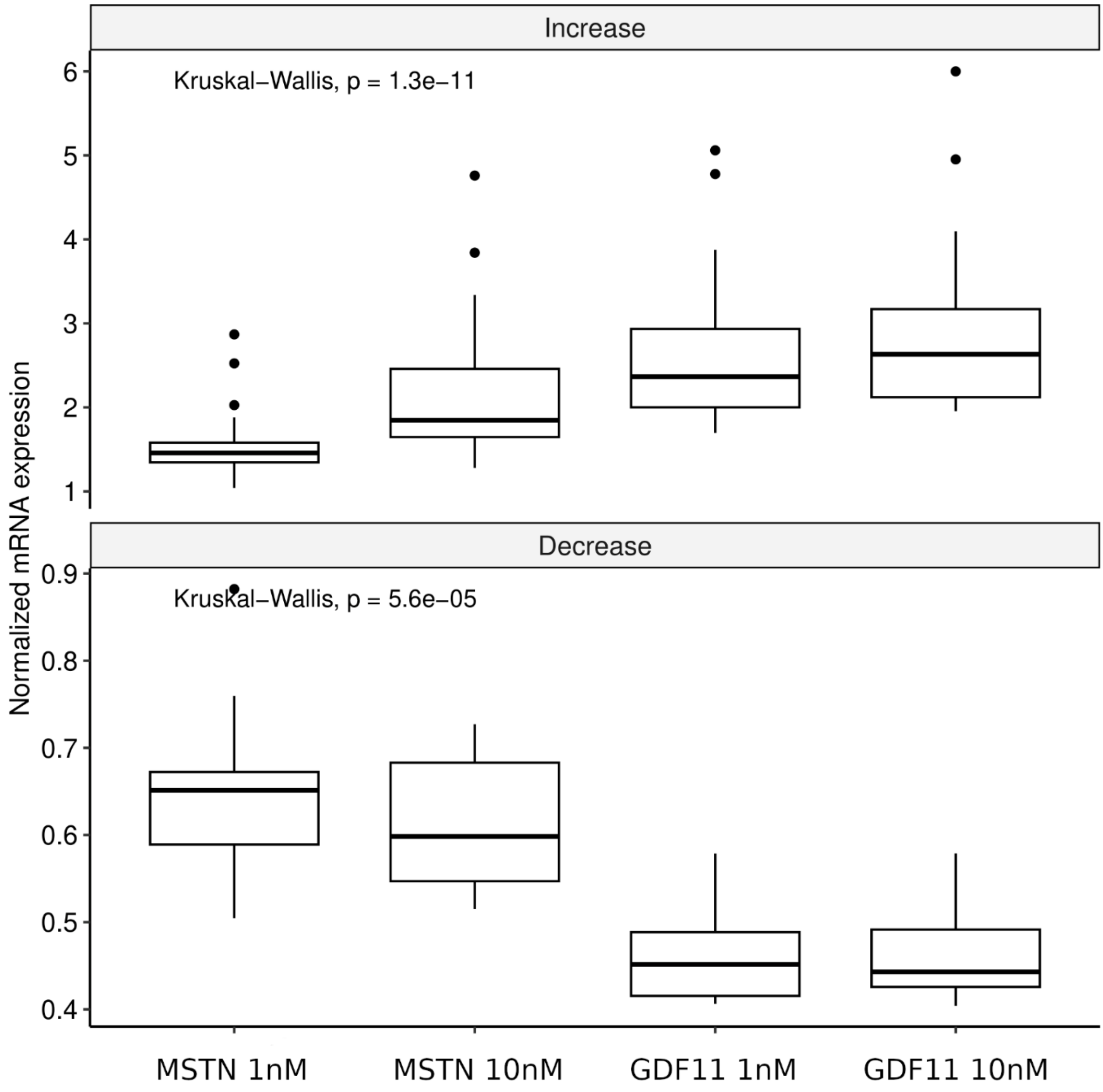

Figure S1 - Boxplots showing genes which were differentially expressed in at least one of the comparisons. The 29 genes which were higher expressed in GDF11 10 nM than in the control are shown at the top. The nine genes were the expression was lower in GDF11 10 nM, are shown at the bottom. The plots are based on normalized values for the four conditions in relation to those of the control.
