# Supplementary material for: Expression profiling by high-throughput sequencing reveals GADD45, SMAD7, EGR-1 and HOXA3 activation in Myostatin (MSTN) and GDF11 treated myoblasts
Source: Genet Mol Biol. 2024 Jul 15;47(2):e20230304. doi: 10.1590/1678-4685-GMB-2023-0304 (PMC11256782; doi:10.1590/1678-4685-GMB-2023-0304)
Supplement: Figure S2 - [file 1415-4757-GMB-47-02-e20230304-s2.pdf]

Genetics and Molecular Biology
https://doi.org/10.1590/1678-4685-GMB-2023-0304

Supplementary Material to "Expression profiling by high-throughput sequencing reveals GADD45, SMAD7, EGR-1 and HOXA3 activation in Myostatin (MSTN) and GDF11 treated myoblasts"

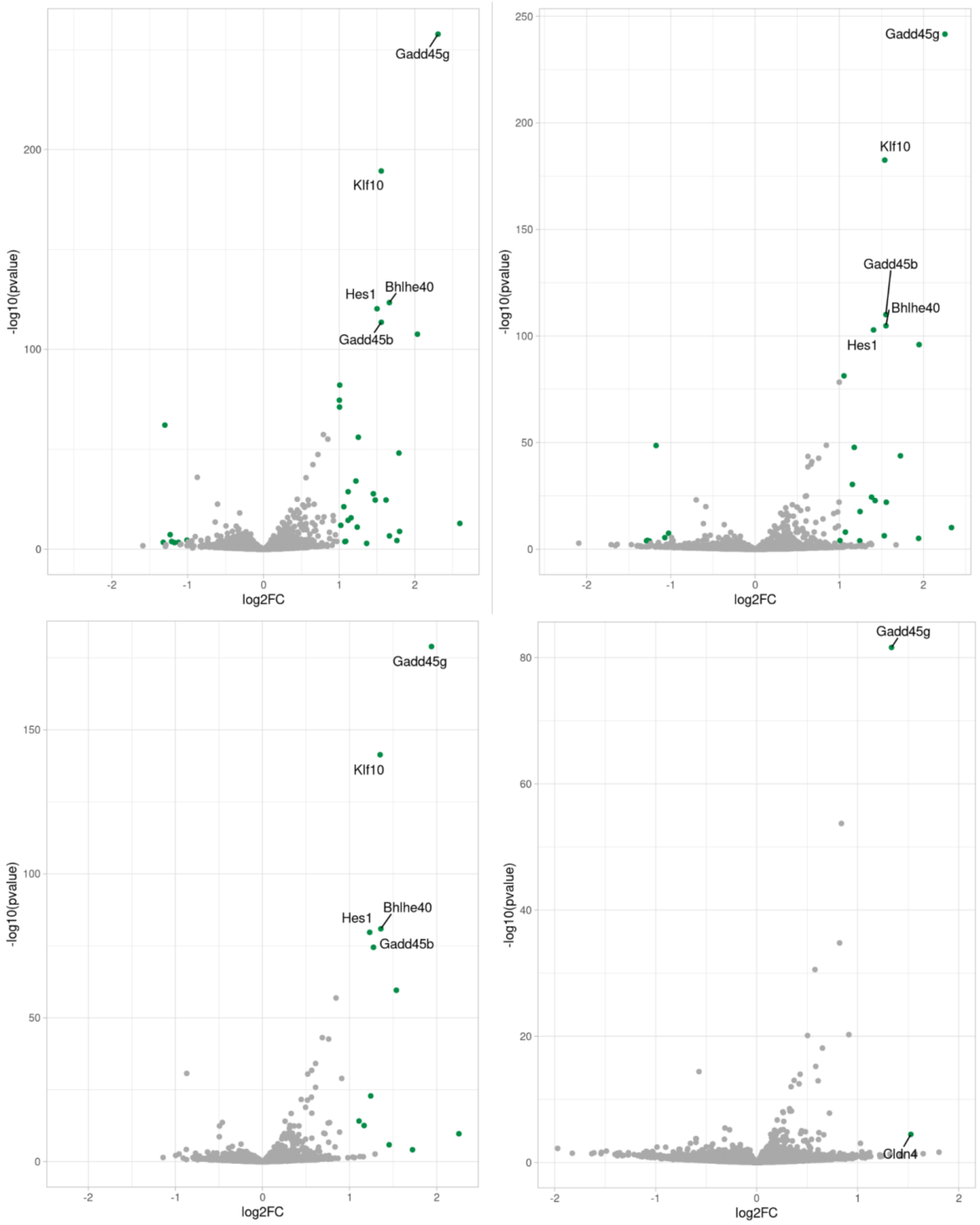

Figure S2 - Volcano plots depicting the expression of genes in GDF11 10 nM, GDF11 1 nM, MSTN 10 nM, and MSTN 1 nM in comparison to the control. Differentially expressed genes are shown as green dots, while other genes are represented as gray dots. In each plot the up to five differentially expressed genes with the lowest p-value were labeled with their corresponding gene symbols.
