# Supplementary material for: Expression profiling by high-throughput sequencing reveals GADD45, SMAD7, EGR-1 and HOXA3 activation in Myostatin (MSTN) and GDF11 treated myoblasts
Source: Genet Mol Biol. 2024 Jul 15;47(2):e20230304. doi: 10.1590/1678-4685-GMB-2023-0304 (PMC11256782; doi:10.1590/1678-4685-GMB-2023-0304)
Supplement: Table S1 - [file 1415-4757-GMB-47-02-e20230304-s3.pdf]

Genetics and Molecular Biology
https://doi.org/10.1590/1678-4685-GMB-2023-0304

Supplementary Material to "Expression profiling by high-throughput sequencing reveals GADD45, SMAD7, EGR-1 and HOXA3 activation in Myostatin (MSTN) and GDF11 treated myoblasts"

Table S1 - Accession Numbers, publically available at the European Nucleotide Archive (ENA) as part as part of study PRJEB57932.

Sequence reads used in this study			
**Title**	**Accession**	**BioSample**	**Readpairs**
GDF11_10nM_A	ERS18229336	SAMEA115285349	22107873
GDF11_10nM_B	ERS18229337	SAMEA115285350	32760081
GDF11_10nM_C	ERS18229338	SAMEA115285351	24078289
GDF11_10nM_D	ERS18229339	SAMEA115285352	30131300
GDF11_1nM_A	ERS18229340	SAMEA115285353	24008272
GDF11_1nM_B	ERS18229341	SAMEA115285354	20284914
GDF11_1nM_C	ERS18229342	SAMEA115285355	23748965
GDF11_1nM_D	ERS18229343	SAMEA115285356	24751582
GDF11_vehicle_10nM_A	ERS18229344	SAMEA115285357	23098428
GDF11_vehicle_10nM_B	ERS18229345	SAMEA115285358	26696536
GDF11_vehicle_10nM_C	ERS18229346	SAMEA115285359	28349452
GDF11_vehicle_10nM_D	ERS18229347	SAMEA115285360	32148537
MSTN_10nM_A	ERS18229356	SAMEA115285369	27574119
MSTN_10nM_B	ERS18229349	SAMEA115285362	29049700
MSTN_10nM_C	ERS18229350	SAMEA115285363	28524155
MSTN_10nM_D	ERS18229351	SAMEA115285364	24165316
MSTN_1nM_A	ERS18229352	SAMEA115285365	27830844
MSTN_1nM_B	ERS18229353	SAMEA115285366	25657178
MSTN_1nM_C	ERS18229354	SAMEA115285367	28713939
MSTN_1nM_D	ERS18229355	SAMEA115285368	28486304

The sequence reads have been submitted to the European Nucleotide Archive (ENA) as part of study PRJEB57932.
